# A Cell-Based Systematic Review on the Role of Annexin A1 in Triple-Negative Breast Cancers

**DOI:** 10.3390/ijms23158256

**Published:** 2022-07-26

**Authors:** Lishantini Pearanpan, Fariza Juliana Nordin, Ee Ling Siew, Endang Kumolosasi, Ezanee Azlina Mohamad Hanif, Siti Fathiah Masre, Eng Wee Chua, Hong Sheng Cheng, Nor Fadilah Rajab

**Affiliations:** 1 Biomedical Science Program, Center for Healthy Aging and Wellness, Faculty of Health Sciences, Universiti Kebangsaan Malaysia (UKM), Jalan Raja Muda Abdul Aziz, Kuala Lumpur 50300, Malaysia; p103277@siswa.ukm.edu.my (L.P.); farizajuliana@gmail.com (F.J.N.); 2 Department of Biotechnology, Faculty of Applied Sciences, UCSI University, Kuala Lumpur 56000, Malaysia; 3 ASASIpintar Program, Pusat Genius@Pintar Negara, Universiti Kebangsaan Malaysia (UKM), Bangi 43600, Malaysia; sieweeling@ukm.edu.my; 4 Biocompatibility and Toxicology Laboratory, Centre for Research and Instrumentation Management (CRIM), Universiti Kebangsaan Malaysia (UKM), Bangi 43600, Malaysia; 5 Faculty of Health Sciences, Universiti Kebangsaan Malaysia (UKM), Jalan Raja Muda Abdul Aziz, Kuala Lumpur 50300, Malaysia; 6 Centre for Drug and Herbal Development, Faculty of Pharmacy, Universiti Kebangsaan Malaysia (UKM), Kuala Lumpur 50300, Malaysia; e_kumolosasi@ukm.edu.my (E.K.); cew85911@ukm.edu.my (E.W.C.); 7 UKM Medical Molecular Biology Institute (UMBI), UKM Medical Centre, Jalan Ya’acob Latiff, Bandar Tun Razak, Cheras, Kuala Lumpur 56000, Malaysia; ezanee.azlina.mohamad.hanif@ppukm.ukm.edu.my; 8 Center for Toxicology and Health Risk Studies, Faculty of Health Sciences, Universiti Kebangsaan Malaysia (UKM), Jalan Raja Muda Abdul Aziz, Kuala Lumpur 50300, Malaysia; sitifathiah@ukm.edu.my; 9 Lee Kong Chian School of Medicine, Nanyang Technological University Singapore, Singapore 308232, Singapore; hscheng@ntu.edu.sg

**Keywords:** Annexin A1, TNBC, metastasis, invasion, proliferation, tumor microenvironment

## Abstract

Triple-negative breast cancer (TNBC) is an aggressive breast cancer subtype that is often associated with a poorer prognosis and does not respond to hormonal therapy. Increasing evidence highlights the exploitability of Annexin A1 (AnxA1), a calcium dependent protein, as a precision medicine for TNBC. To systematically summarize the role of AnxA1 and its associated mechanisms in TNBC, we performed data mining using three main databases: PubMed, Scopus, and Ovid/Medline. The papers retrieved were based on two different sets of key words such as “Annexin A1” or “Lipocortin 1” and “Breast cancer” or “TNBC”. A total of 388 articles were identified, with 210 chosen for comprehensive screening and 13 papers that met inclusion criteria were included. Current evidence from cell culture studies showed that AnxA1 expression is correlated with NF-κB, which promotes migration by activating ERK phosphorylation. AnxaA1 also activates TGF-β signaling which upregulates MMP-9 and miR196a expression to enhance epithelial-mesenchymal transition and migratory capacity of TNBC cells. AnxA1 can steer the macrophage polarization toward the M2 phenotype to create a pro-tumor immune environment. Existing research suggests a potential role of AnxA1 in the metastasis and immune landscape of TNBC tumors. Preclinical and clinical experiments are warranted to investigate the feasibility and effectiveness of targeting AnxA1 in TNBC.

## 1. Introduction 

Breast cancer is the world’s most prevalent cancer among women [1] with 2,261,419 new cases and 684,996 deaths, according to GLOBOCAN 2020 [2]. TNBC accounts for about 15–20% of all breast cancer subtypes [3,4,5]. It is clinically characterized by the lack of main receptors of breast cancer; estrogen receptor (ER) and progesterone receptor (PR), as well as the amplification of human epidermal growth factor 2 (HER2) [3,6,7]. It is associated with rapid growth, distant metastasis, invasiveness, high risk of relapse and poor prognosis [8,9]. Due to the lack of receptors, TNBC does not benefit from hormonal-based therapies, conventional chemotherapy is the only treatment available as the mainstream therapeutic approach for patients with TNBC, despite the considerable side effects and suboptimal outcomes [3,9]. To reduce the burden of TNBC, it is critical to establish optimum therapeutic options for the treatment of early TNBC [9]. Recently, three targeted therapies for TNBC have been approved by US Food and Drug Administration, including the Poly (ADP-ribose) polymerase (PARP) inhibitors olaparib and talazoparib for germline BRCA mutation-associated breast cancer and the checkpoint inhibitor, atezolizumab in combination with nab-paclitaxel for programmed death-ligand 1 positive TNBC [10,11]. However, understanding the complexity and heterogeneity of the disease, more personalized targeted treatment options are still needed to treat TNBC patients. Therefore, we must continue to explore potential predictors responsible for TNBC progression.

Annexin A1 (AnxA1) also known as lipocortin-1 [12] is a 37kDa annexin superfamily, which is calcium and phospholipid dependent [13]. AnxA1 engages in anti-inflammatory effects where it modulates innate immune cell activity, such as neutrophils and macrophages [14], cell differentiation regulation, proliferation, and apoptosis [15]. AnxA1 mediated activity is mainly through the activation of formyl peptide receptors (FPRs) which are the only known receptor of AnxA1 [16,17,18]. To facilitate the externalization and localization of AnxA1, the protein must be activated by glucocorticoids [19].

AnxA1 plays varying roles in tumorigenesis [20]. A recent systematic review concluded a strong association of AnxA1 overexpression with hallmarks of cancer, including uncontrolled proliferation, metastasis, lymphatic invasion and drug resistance according to studies on pancreatic, hepatic, gastric, skin and colorectal cancers [19] The aggressive behaviors are typically found in TNBC, highlighting a potential implication of AnxA1 in the oncogenesis of the poor-prognosis subtype. Thus, to build on the existing knowledge, our systematic review zooms into the role of AnxA1 and its related mechanisms in TNBC to explore new therapeutic options for precision oncology in TNBC. 

## 2. Methods

Preferred Reporting Items for Systematic Reviews and Meta-Analysis (PRISMA) were used to design the study [21]. Since this systematic review primarily focuses on in-vitro studies, we did not register the systematic review in PROSPERO, which focuses more on human studies and animal studies.

### 2.1. Search for Relevant Studies

A systematic search was conducted based on three online electronic databases namely PubMed, Ovid/Medline, and Scopus for all relevant articles published from January 2000 until January 2020. The following keywords were used to search the databases: “Annexin A1” or “Lipocortin-1” and “Breast cancer” or “Triple-Negative Breast Cancer”. Those articles were imported to Mendeley software and duplicates were removed. The language of publication was decided to be English only.

### 2.2. Selection of Articles

Abstracts and titles of the retrieved papers were screened (first screening), and relevant articles were chosen based on eligibility criteria. Then the full texts of selected articles (second screening) and the articles that fit the inclusion and exclusion were included in this review. The eligibility for inclusion was based on “AnxA1 in TNBC” including in-vitro studies. Concurrently, studies that are not relevant to AnxA1, TNBC, limited data on the biological outcome, poorly developed methodologies, biased outcomes and insufficient sample size are all ruled out. Two independent researchers carried out these procedures, and the differences (between the two researchers) were compared, discussed and the agreement was reached accordingly. 

## 3. Results and Discussion

### 3.1. Study Selection

**Figure 1:**
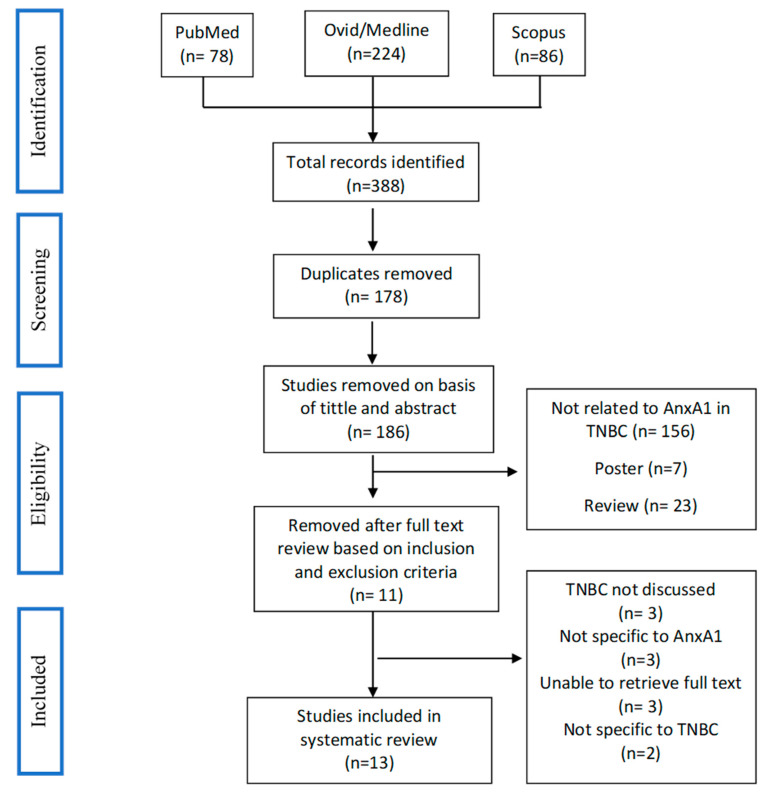
Search strategy and study selection flow chart.

A total of 388 articles were identified, after removal of duplications, 210 articles were chosen for comprehensive screening and 13 articles that met inclusion criteria as described in the “Methods” were included. Figure 1 shows the study selection process using the PRISMA flow diagram.

### 3.2. Data Extraction and Synthesis

Data extracted included information of authors, name of cell lines, culture conditions whether it is comparable to other studies, study findings, and significant statistical value. A qualitative synthesis of data was employed to summarize the information obtained from the selected articles. A meta-analysis (or quantitative analysis) was not conducted due to insufficient homogeneous outcomes from the selected articles.

### 3.3. Quality Assessment

The Quality Assessment (QA) methods used to determine the likelihood of bias associated with cell culture studies were carried out using criteria recommended by WCRF/UoB [22,23]. For each parameter, the score range 0–6 was assigned (a score of ‘1’ was given to ‘yes’, ‘0’ to parameters that ‘are not met’ or ‘not reported’. Based on the average score, studies were reported as low (0–2), moderate (3–4), or (5–6) quality in Table 1. This bias assessment was not used to evaluate the inclusion and exclusion criteria. A summary of the bias assessment is useful to make a conclusion as to whether the research included supporting the biological plausibility of the investigated possible mechanism. 

### 3.4. AnxA1 Is a Subtype-Specific Markers in TNBC

In breast cancer, AnxA1 expression shows a distinct subtypes specificity [34]. Compared to other subtypes, TNBC consistently expresses higher levels of AnxA1 which correlates with the disease severity as well as poor prognosis [35]. 

AnxA1 is highly expressed in the most aggressive, metastatic, and invasive cell line, MDA-MB 231 (TNBC cell line), as compared to MCF-7 (non-TNBC cell line) and MCF-10A (human mammary cell line) [32]. In the previous study, TNBC cell lines (MDA-MB 231, MDA-MB 157, MDA-MB 436, MDA-MB 468, HCC70, BT-549, and Hs578T) expressed a high level of AnxA1 [30,33,36]. These cells were known as mesenchymal-like and identified as breast cancer cells of cytokeratin (CK) 5+/ER-basal-like [26]. Interestingly, AnxA1 was not found in MCF-7, T47D, SKBR3, and ZR751 [13,15,20,32]. Under resting conditions, cells have high levels of AnxA1 in the cytoplasm; after activation, AnxA1 is transported to the cell surface and secreted [37]. The high amount of AnxA1 found in supernatants was mostly in its 33 and/or 35.5 kDa cleaved form [30,32], which is the key driver of tumor aggressiveness. Not only in its cleaved form but higher expression of full-length AnxA1 (37 kDa) was also observed in MDA-MB 231 cells as compared to MCF-7 and MCF-10A [32]. 

Clinically, patients with TNBC expressing a high level of AnxA1 and have a poorer survival rate as compared to patients with a low level of AnxA1 [37]. AnxA1 is associated with TNBC cell invasion as well as migration [13,30]. AnxA1 expression is more likely to be elevated in breast metastases, especially those seen in regional lymph nodes, the lung, and the liver [18]. Since AnxA1 in TNBC cells is recognized for its highly aggressive characteristics and therefore blocking this signal is considered a therapeutic strategy for TNBC management. However, understanding the heterogeneity of TNBC with different molecular subtype classification by Lehmann et al., including basal-like (BL) 1, BL2, mesenchymal, mesenchymal stem-like, immunomodulatory, and luminal androgen receptor [38,39], AnxA1 expression in cell lines representing immunomodulatory and luminal androgen receptor subtypes has to be studied so that the result reflects the whole TNBC spectrum.

### 3.5. AnxA1 Induces Epithelial-Mesenchymal Transition in TNBC

In cancer cells, epithelial-mesenchymal transition (EMT) is characterized by progressive acquisition of mesenchymal and pro-migratory features with heightened resistance to pro-apoptotic signals and chemotherapeutic assaults [36]. EMT is a key feature of epithelial tumors and plays pivotal role in metastasis [18]. Kang and colleagues investigated the role of AnxA1 in the migration and invasion of TNBC cells [13]. The cell line with high AnxA1 expression, was invasive whereby cells with low or no AnxA1 expression exhibited a poor migratory profile [13,15,40]. The involvement of AnxA1 has been correlated with matrix metalloproteinase 9 (MMP-9) gene expression and its regulation of intrinsic MMP-9 gene transcription and proteolytic activity [13]. Breast cancer cells treated with siAnxA1 have suppressed NF-κB activity, which results in downregulation of MMP-9 expression and impaired cell migration [13]. Among all the MMPs, MMP-2 and MMP-9 (gelatinase B) are highly associated with aggressive, metastatic breast cancer and lung cancer [41,42] as they can cause proteolytic degradation of the extracellular matrix [20,41] and basement membrane [41]. Different biochemical simulators, including growth factors, cytokines, and phorbol 12-myristate 13-acetate (PMA), regulate MMP-9 by activating different intracellular signaling pathways [43]. Studies have shown that MMP2 and MMP9 are crucial for tumor cell invasiveness, and MMP9-mediated invasion inhibition suppresses tumor cell metastasis [13]. As a result, there is a clear association between AnxA1 expression and both cell migration as well as invasion. 

The effect of AnxA1 knockdown on cancer cell migration has also been investigated in past studies. Interestingly, results showed that AnxA1 knockdown cells had significantly shorter migration distances which were directly mediated through the mammalian target of the rapamycin (mTOR) signaling pathway [20]. 

Activation of mTOR-S6 signaling is correlated with AnxA1 in TNBC. Activation of this pathway has been linked to the capacity of cancer to generate a new vasculature network, inflammation, and cancer cell survival [44]. Ribosomal protein S6 RPS6 and S6 kinase (S6k), the downstream effector proteins of the mTOR pathway are required for the metastatic behavior and progression of cancer [20,44] via F-actin modification and MMP-9 up-regulation [20]. S6K is highly expressed in lung and ovarian cancers, and the expression of S6K is associated with poor breast, kidney, and hepatocellular carcinoma prognosis [45] and RPS6, in addition, has been suggested as a potential cancer biomarker [44]. Downregulation of RPS6 alone could significantly reduce the proliferation of TNBC [44]. Furthermore, RPS6-knockdown in non-small cell lung cancer (NSCLC) has been shown to regulate the EMT-related proteins where MMP-9, MMP-2, Vimentin and N-cadherin have been downregulated and E-cadherin has been upregulated [44]. A similar trend in the regulation of vimentin and E-cadherin (hallmarks of EMT) [20] has been observed in a study that examined the DC-STAMP domain containing 1-antisense 1 (DCST1-AS1) in promoting EMT via AnxA1 [33].

High AnxA1 was also linked to Cathepsin D (CatD) expression, with CatD expression in AnxA1 knockdown MDA-MB 231 cells being significantly lower than in normal MDA-MB 231 cells [32]. Cathepsins are a protease superfamily made up of 16 members of aspartyl, serine, and cysteine proteases that are highly expressed in various tumors and associated with metastasis [32,46] and currently CatD is being explored as a potential TNBC marker. Pepstatin A (CatD inhibitor), can reduce the cell growth, invasiveness, and migratory potential of MDA-MB 231 cells. Mechanistically, AnxA1’s N-terminal peptides activate the formyl peptide receptors (FPRs), namely FPR1 and FPR2 [32], which are the only known receptors of extracellular AnxA1 [18]. By inhibiting CatD catalytic properties, AnxA1 mediated signaling pathways are hindered. Aside from breast cancer, the AnxA1 and FPR complex has been associated with the invasive nature of numerous cancers, including colon, rectal, gastric, prostate, pancreas, melanoma, and esophageal squamous cell carcinoma [47,48,49].

In addition, in MDA-MB 231 cells, FPR1 was reported to be 4-fold higher than human monocytes while FPR2 was 8-fold higher than MCF-7 cells [27]. The activation of the AnxA1 receptor (FPR2) induces cell proliferation of MDA-MB 231 cells which is attenuated by FPR2 antagonists (WRW4 and Boc2) and AnxA1 knockdown [27]. Additionally, mitogens can promote AnxA1 expression, subsequently activating FPR2 and causing further mitogenesis in TNBC via cyclin D1 upregulation [27]. Such mitogen-induced cell proliferation in MDA-MB 231 is inhibited by FPR2 siRNA silencing, clearly showing the pivotal role of AnxA1-FPR2 signaling in cell cycling of TNBC [27]. 

On the other hand, FPR1 inhibition in MDA-MB 231 showed a positive association in primary tumors specifically on the expression pattern of AnxA1 and its receptor, FPR1 [30]. Moreover, FPR1 inactivation by Cyclosporin H (CsH) and Cyclosporin A (CsA) drugs can decrease cytosolic calcium levels, increase IL-6 secretion, and reduce the extracellular signal-regulated kinase (ERK) phosphorylation, leading to suppressed cell migratory and invasive capability [30]. Therefore, inhibition of FPR1 signaling by CsH and CsA could also be developed as a potential therapeutic strategy for TNBC management. 

4T1, a highly metastatic and invasive mouse TNBC cell line, exhibited increased growth and invasive capability co-cultured with AnxA1^+/+^ M2 polarized macrophages, which suggests that macrophages can potentially influence the tumor cell proliferation and migration dependent on AnxA1 [28]. Treatment of macrophages with conditioned media of the breast tumor, especially 4T1, can skew the kinetic profiles to the M2 subset (alternatively activated M2) [28]. AnxA1 is also required for tumor-associated macrophage (TAM) polarization to adopt an M2 phenotype which enhances the breast cancer immunosurveillance escape, migration, and metastasis [16,28]. Tumor-associated macrophages (TAM) are found at a very high level in many tumors, such as ovarian, breast, and pancreatic cancer [50]. TAMs especially M2 macrophages facilitate cancer metastasis through multiple mechanisms including angiogenesis promotion, tumor growth activation, tumor cell migration, and invasion [51,52,53]. M2 phenotype is pro-tumor as its functions include cell division, metastasis, and tumor cell survival [53], and AnnexinA1 is required for the macrophage polarization to an M2 phenotype which promotes the tumor growth and invasion [28].

AnxA1 overexpression together with SDF-1α (stromal-derived factor 1α or also known as chemokine CXCL12) treatment exhibits a significant increase in migration, and together with CXCR4 induces concentration-dependent migration [25]. CXCR4 is a downstream target of NF-κB. In response to chemokine CXCL12, AnxA1 significantly modulated the CXCR4-mediated migration of breast cancer cell lines, suggesting its significance in tissue-specific migration of breast cancer cells [25]. CXCL12 is expressed in organs serving at major sites of metastasis of breast cancer, such as the bone and lung, while its CXCR4 receptor is well expressed in cells of metastatic breast cancer [40]. Intracellular signaling is induced by CXCL12 binding to CXCR4 through a variety of pathways that stimulate chemotaxis, cell survival and/or proliferation, intracellular calcium increase, and gene transcription signal [54].

Targeting AnxA1 that regulates EMT and its related proteins mainly matrix metalloproteases, vimentin and E-cadherin may be a promising marker in inhibiting metastasis, invasion and proliferation of TNBC. The role of AnxA1 in inducing TNBC cell migration, invasion, and proliferation has been summarized in Table 2.

### 3.6. AnxA1 Diminishes Sensitivity of TNBC Cells to Apoptotic Signals 

As previously stated, Cathepsin D (CatD) is important not just in migration, invasion, and proliferation, but also in preventing apoptosis in TNBC via AnxA1 cleavage [32]. CatD protects MDA-MB 231 cells from apoptosis and autophagy by enhancing the cleavage of AnxA1 into smaller fragments (35.5 KDa). Not only that, FPR1 inhibition by Cyclosporin H (CsH) and Cyclosporin A (CsA) induced cell cycle arrest and early apoptosis after 24-h treatment in MDA-MB 231 cells without affecting the cell line of MCF-7 and MCF-10A [30]. Additionally, it has been shown that AnxA1 secretion induces autocrine signaling in MDA-MB 231 cells via FPR1 [30]. Therefore treatment (Pepstatin A) that keeps AnxA1 (37 KDa) intact, reduces aggressive behavior in MDA-MB 231 cells and promotes apoptosis and autophagy [32].

One of the hallmarks of cancer is the inhibition of apoptosis [8] and it is regulated by the RIPK1 pathway, which can, in turn, modulate NF-κB, NF-κB is a transcription factor that plays an important role in desensitizing cells to apoptosis by decreasing reactive oxygen species (ROS) and antagonizing the p53 protein [55]. Since AnxA1 expression is significantly associated with NF-κB, thus its modulation either directly or indirectly can disrupt RIPK1 signaling, affecting apoptosis and leading to cancer progression [19]. 

The role of AnxA1 in desensitizing cells to apoptosis of TNBC has been summarized in Table 3.

### 3.7. AnxA1 and Multidrug Resistance (MRD)

The first evidence for the role of AnxA1 in the multidrug resistance of MDA-MB 231 cells has been reported [29]. As compared to MCF7 (AnxA1^−^), MDA-MB 231 (AnxA1^+^) had a 3 to 20-fold rise in resistance to Adriamycin, Melphalan, and Etoposide. In tumor cells, AnxA1 depletion increases their susceptibility to anti-cancer drugs [29]. However, the exact mechanism by which AnxA1 exerts drug resistance to the anti-tumor drug is still unknown [29].

DC-STAMP domain-containing 1-antisense 1 (DCST1-AS1) is known to regulate the drug resistance of BT-549 cells to doxorubicin and paclitaxel by binding to AnxA1 [33]. Typically, DCST1-AS1 knockdown cells have increased susceptibility to doxorubicin and paclitaxel. However, such drug susceptibility due to the DCST1-AS1 knockdown is lost when AnxA1 is overexpressed. 

Resistance to multidrug is one of the biggest challenges that is faced to treat TNBC. The findings together suggest a profound implication of AnxA1 in the aggressiveness of TNBC and susceptibility to treatment that has yet to be thoroughly understood. Hence, modulating the expression of AnxA1 can be a potential strategy to overcome multidrug resistance. 

### 3.8. Immunomodulatory Role of AnxA1 in TNBC

4T1 breast cancer cells exhibit an AnxA1-dependent M2 macrophage polarization that promotes tumor growth, invasion, and migration [28]. 4T1 (AnxA1^+^) or Ac2-26 (AnxA1 peptide) enhance the ERK and NF-κB activation via formyl peptide receptor 2 (FPR2) and ERK signaling, downstream of AnxA1 which is required for macrophages to polarize into an M2 phenotype [28]. Additionally, high expression of chemokine C-C motif chemokine ligand 5 (CCL5) is also associated with AnxA1 to promote macrophage polarization. Chemokine CCL5 expression is elevated in tissues and plasma of patients with advanced-stage breast carcinoma [28]. Macrophages treated with recombinant CCL5 displayed an increased expression of AnxA1, revealing a new linkage between AnxA1 and CCL5 chemokine which could modulate the intratumoral immune landscape.

Furthermore, a high level of AnxA1 in TNBC induces Treg cell-mediated immunomodulation, where inhibiting AnxA1 can weaken Treg cells resulting in the reduction of tumor size in the in-vivo model [37]. RNA-seq reveals that inhibiting AnxA1 reduces granzyme A expression in Treg cells which can dampen natural killer cells and cytotoxic T lymphocytes. Moreover, the expression of key Treg surface markers such as IL2RA (CD25), CCR8, and PDCD1 has diminished [37]. AnxA1 may exert a profound regulator role in onco-immunity in the tumor microenvironment of TNBC, however, these warrant further investigation involving knockdown studies using a co-culture model.

### 3.9. Mechanism of Action Related to AnxA1

Silencing AnxA1 in MDA-MB 231 cells decreased NF-κB DNA binding, which results in a reduction of IkB kinase (IKK), IκBα, and p65 phosphorylation [25]. Additionally, NEMO and receptor-interacting protein (RIP) are associated with AnxA1 in MDA-MB 231 cells. Disruption of these interactions is disrupted, leading to the inactivation of NF-κB [25]. The NEMO-ANXA1-RIP complex facilitates a survival response through the upregulation of NF-κB. Not only that, AnxA1 knockdown in MDA MB-231 cells could reduce the upregulation of uPA, a target gene of NF-κB which has significant involvement in metastasis [25]. 

AnxA1 is also involved in the regulation of microRNAs which are a group of non-coding RNAs that have been demonstrated to control many of the genes involved in cellular processes such as proliferation, differentiation, and apoptosis [24]. AnxA1 inhibited the transcription of miR196a which in turn induced a negative feedback loop to enhance breast cancer proliferation, migration, and metastasis [31]. MiR196a is an oncogenic miRNA that is used as a diagnostic biomarker for many cancers including laryngeal, colorectal, and glioma cancers. Additionally, high expression of AnxA1 leads to a decrease in miR26b^*^ and miR562 which enhances NF-κB activity. Conversely, MCF-7 cells, which have lower AnxA1 levels, have a high level of miR26b^*^ and reduced NF-κB activity. Therefore, existing evidence supports a regulatory role of AnxA1 on NF-κB activity via miRNA modulation.

In TNBC cell lines, a higher endogenous level of AnxA1 is associated with higher EGFR, c-Met, and AKT [20]. Additionally, AnxA1 knockdown cells have reduced mTOR and protein S6 phosphorylation [20] but a 1.8-fold times increase in the phosphorylation of a stress-induced protein, AMPKα [20,56]. How AnxA1 interacts with AMPK to control the mTOR signaling pathway remains unclear. One possible mechanism is through the activation of pATM, which is involved in stress response and can activate AMPK [20]. The mammalian target of rapamycin (mTOR) is a major regulator of cell growth in normal circumstances, however, mTOR signaling in tumor cells is alternatively activated to stimulate metastasis and invasion [57]. This AnxA1-AMPK-mTOR-cell migration could explain why AnxA1 levels are linked to a poor prognosis in TNBC.

On the other hand, AnxA1 knockdown inhibited the Smad2 phosphorylation, Smad 4 nuclear translocation, and Smad 3 and 4 driven transcriptional activity caused by transforming growth factor β (TGF-β) [26]. By regulating TGF-β and actin reorganization, AnxA1 promotes epithelial-mesenchymal transition (EMT) and migration of breast cancer cells [26]. Phosphorylation of downstream mediators of TGF-β (Smad2 and Smad3) forms a complex with Smad4 that translocates into the nucleus to modulate gene transcription [58]. Another study showed that the DC-STAMP domain-containing 1-antisense 1 (DCST1-AS1) which is highly expressed in TNBC (MDA-MB 231 and BT-549 cells) directly binds to AnxA1 and promotes EMT through TGF-β/Smad signaling [33]. TGF-β signaling plays a very important role in tumor initiation and progression. TGF-β will increase the tumor growth and invasiveness of breast cancer cells by activating the expression of MMP-2 and MMP-9 [33].

Apart from the different mechanisms explained above, AnxA1 primarily promotes the activation of NF-κB signaling which is a mediator of AnxA1-induced breast cancer cell migration [18]. Nuclear translocation of NF-κB triggers cell proliferation genes (cyclins/CDKs), thus promoting cell growth. Additionally, there is a positive association between NF-κB activation and increased expression of anti-apoptotic members of the Bcl-2 family, resulting in resistance to apoptosis [59]. In HER2-positive tumors and TNBC, the constitutive activation of NF-κB is responsible for cell proliferation, angiogenesis, and inhibition of apoptosis [8]. MDA-MB 231 cells have impaired cell proliferation when NF-κB signals are blocked by mutant IκB [8]. Additionally, it also induced an epithelial-mesenchymal transformation and promote migration capability, involving TGF-β-dependent signaling. Apart from that, NF-κB-induced stimulation of MMP-9 production is inhibited by AnxA1 knockdown in TNBC cells preventing the subsequent cancer cell migration [13]. 

**Figure 2:**
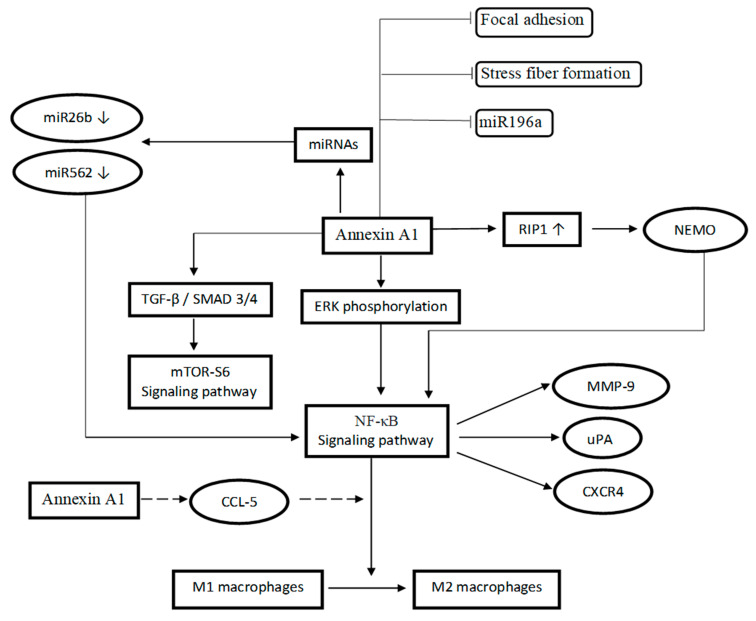
Schematic illustration of proposed AnxA1 mechanism of action via different signaling pathways.

The mechanism of action related to AnxA1 has been summarized in Table 4 and a schematic diagram has been developed to provide a broad picture of the mechanism of action via different pathways influenced by AnxA1 (Figure 2). Although AnxA1 has been well implicated in a number of TNBC-related mechanisms, however additional research is required to fully comprehend how this protein interacts with common signaling pathways of TNBC. Rubinstein et al. described AnxA1 as an unknown regulator of Wnt/β-catenin signaling and a vital protein in colorectal cancer progression [60]. However, the expression of β-catenin in glioma cells was not affected by AnxA1 expression [61]. Given that AnxA1 is closely associated with the targeted genes of this pathway, MMP-9 and MMP-2 [13] and cyclin-D1 [27], it is possible that AnxA1 also plays a role in the regulation of Wnt/β-catenin signaling in TNBC. Targeting this signal is equally crucial as it plays a significant role in the progression and metastatic nature of TNBC by increasing the MMP-7 expression [62,63].

Based on the studies presented, the aberrant pathways (mTOR, TGF-β and NF-κB signaling) that are affected by the direct or indirect effect of AnxA1 contributes to the progression of TNBC. With the extended involvement of AnxA1 in various signaling, we believe that it has the potential to be developed as a novel multi-targeted protein to inhibit dysregulated pathways in TNBC. However, extensive study is needed to fully understand AnxA1′s role in other TNBC-related key pathways such as Notch, mitogen-activated protein kinase (MAPK) and Hedgehog signaling [8].

## 4. Conclusions

Current evidence suggests a unique role of AnxA1 in the progression of TNBC. It should be noted that most findings are derived from TNBC cell lines, whereas in vivo animal experiments and clinical data are lacking. Furthermore, TNBC is a heterogenous breast cancer that can be subclassified into six subtypes, but most of the mechanistic studies on AnxA1 covered only the mesenchymal, BL1 and BL2 TNBC cell lines. Conceivably, the three subtypes represent about 70–80% of all TNBC cases [8], allowing us to infer a decent association between AnxA1 and TNBC. Future mechanistic studies should focus on the role of AnxA1 in the under-represented TNBC molecular subtypes. Disease models with better clinical relevance such as mammosphere culture and mouse tumor xenografts should be used to interrogate the targetability of AnxA1 in highly heterogenous TNBC. 

AnxA1 is well-implicated in the modulation of tumor inflammation via NF-κB activation. It can also remodel the macrophage polarization to favor alternatively activated macrophages or tumor-associated macrophages which contributes to the aggressiveness of TNBC. However, the underpinning mechanism remains largely elusive. Further investigation should employ the TNBC-macrophage co-culture models to explore the AnxA1-mediated tumor-immune crosstalk, which may reveal the exciting role of AnxA1 in the tumor microenvironment.

Additionally, AnxA1 exhibits a significant involvement in the epithelial-to-mesenchymal transition process via the modulation of TGF-β signaling and extracellular matrix remodeling enzymes. Such activity can confer profound chemoresistance, which is a typical feature of TNBC. Interestingly, AnxA1 ablation restores the sensitivity of colorectal and pancreatic cancers to chemotherapeutic agents through the inhibition of the drug efflux pump, p-glycoprotein [64,65]. Hence, it may be worthwhile to examine the intrinsic functionality of AnxA1 on drug efflux pumps in TNBC. The results can potentially underscore its practicality as an adjunctive targeted therapy to potentiate chemotherapeutic agents in TNBC treatment. Taken together, AnxA1 is implicated in the aggressive behaviors of TNBC, including proliferation, metastasis, invasion, pro-survival, and chemoresistance. The underlying mechanisms are linked to the activation of NF-κB and TGF-β signaling which are key pathways for tumor inflammation and epithelial-to-mesenchymal transition. AnxA1 also promotes the alternative activation of macrophages, suggesting a role in the formation of an immunosuppressive TNBC tumor microenvironment. Although existing evidence supports the pro-tumorigenic role of AnxA1 in TNBC, further validation using preclinical animal models and different subtypes of TNBCs is warranted.

## Figures and Tables

**Table 1:** Quality assessment of in vitro studies (cell culture) (adapted from WCRF/UoB recommendations).

Study	Source ^a^	Experimental Design				Selective Reporting ^f^	Total
		Culture Conditions ^b^	Replicates ^c^	Controls ^d^	Multiple Cell lines ^e^		
[24]	1	1	0	1	0	1	4
[20]	1	1	1	1	1	0	5
[25]	1	1	1	1	1	1	6
[26]	1	0	1	1	1	1	5
[13]	1	1	1	1	1	0	5
[27]	1	1	1	1	1	1	6
[28]	1	1	1	1	0	1	5
[15]	1	1	0	1	1	1	5
[29]	1	1	1	1	1	1	6
[30]	1	1	0	1	1	0	4
[31]	1	1	0	1	1	1	5
[32]	1	1	1	1	1	1	6
[33]	1	1	1	1	1	1	6

Studies receive a score of 0 or 1 for each of the following parameters (a score of 0 was given due to lack of fulfillment of the criteria or failure to report): ^a^ Every cell line are validated independently; ^b^ culture conditions are similar to other studies; ^c^ adequate biological and technical repeats are performed; ^d^ proper controls included; ^e^ different cell lines are used; ^f^ all experiment results are included.

**Table 2:** The role of AnxA1 in inducing epithelial-mesenchymal transition in TNBC.

Cell Line(s)	Origin	Findings	Study
MDA-MB 231	Human breast cancer	24 h Pep A (1 μm and 10 μm) treatment impaired MDA-MB 231 cell proliferation. (*p* < 0.01) Treatment of MDA-MB 231 cells with PepA 1 μm reduced the percentage of invasive cells to 40.75%, whereby only 15% of MDA-MB 231 cells with PepA 10 μm were able to invade matrigel relative to control (100% invaded cells) (*p* < 0.01) 24 h treatment with PepA decreased the cell migration of MDA-MB 231 cells (Scale bars = 200 µm)	[32]
MDA-MB 231	Human breast cancer	Both CsA (1 µm) and CsH (0.1 µm) treatment for 24 h decreased both invasive and migratory capacity of MDA-MB 231 cells and those properties were also diminishing in shAnxA1 MDA-MB 231 cells. Whereby treatment with Ac2-26 (10 µm) increased both invasive and migratory property. (*p* < 0.05)	[30]
4-T1	Mouse mammary tumor	4T1 cells co-cultured with AnxA1^+/+^ M2 macrophage (polarized for 24 h with IL4) showed increased proliferation and invasion compared with the control cells (non-polarized media) (*p* < 0.01)	[28]
MDA-MB 231	Human breast cancer	si-AnxA1 (10 nM) and si-AnxA1 (30 nM) transfected MDA-MB 231, resulted in a significant decrease in migratory ability as a result of decreased in percentage of wound healing. (*p* < 0.05). Whereby, the si-AnxA1 transfected as the stated amount for 36 h decreased the invasiveness of MDA-MB 231 cells	[13]
MDA-MB 231	Human breast cancer	The invasion ratio in cells treated with both AnxA1siRNA-1 (*p* < 0.05) and siRNA-2 (*p* < 0.01) was significantly reduced	[15]
MDA-MB 468 MDA-MB 436	Human breast cancer	AnxA1 knockdown cells had significantly shorter migration distance (average 21% to 63%) compared to control cells. (*p* < 0.05) Significant increase about 1.8 to 3.9-fold in cell migration was observed in mTOR transfected cells as compared to control. (*p* < 0.001)	[20]
MDA-MB 231	Human breast cancer	Overexpression of AnxA1 showed significantly increased migration of MDA-MB 231 cells to SDF1α (100 µg/mL) treatment in a concentration dependent manner. (*p* < 005) SDF1α-induced migration significantly decreased in AnxA1 knockdown cells.	[25]
MDA-MB 231	Human breast cancer	Increase in cell number stimulated by FCS (5% *v*/*v*), LXA_4_ (100 nM) and AnxA1_2-26_ (1 µM) were decreased by Boc2 (100 nM) and WRW4 (1 µM). (*p* < 0.05) Cell number (%) increase induced by estradiol and FCS were decreased by FPR1 siRNA transfection (*p* < 0.05) whereby FPR2 siRNA transfection inhibited cell number increase (%) by all mitogens used in MDA-MB 231 cells. (*p* < 0.05) Mitogens used: FCS (5% *v*/*v*), LXA_4_ (100 nM), WKYMVm (10 nM) and EGF (300 pM) (*p* < 0.05)	[27]

**Table 3:** Role of AnxA1 in protecting TNBC cells from apoptosis.

Cell Line (s)	Origin	Findings	Study
MDA-MB 231	Human breast cancer	CatD-induced inhibition of AnxA1 cleavage induces apoptotic cell death in 57 percent of TNBC cells (PepA 1 μm) to 72.8 percent (Pep 10 μm). MDC-labelled vacuoles with 35.0 and 78.40 fluorescence intensities were observed in TNBC cells treated for 24 h with Pep A 1 μm and 10 μm, respectively	[32]
MDA-MB 231	Human breast cancer	CsH (1μm) and CsA (5 μm) treatment resulted in an increase in cells stacked in the G0/G1 phase and a decrease in the percentage of cells in the S and mitosis phases. After 24 h of treatment, CsH (10 μm) induced early apoptosis in 15.8% of MDA-MB 231 cells	[30]

**Table 4:** Mechanisms related to AnxA1.

Cell Line(s)	Origin	Findings	Study
MDA-MB 231	Human breast cancer	Cells treated with CsH (0.1 µm) and CsA (1 µm) increased the IL-6 (pg/mL) secretion and decreased with treatment of Ac2-26 (10 µm) (*p* < 0.01) CsH (0.1 µm) and CsA (1 µm) reduced cytosolic calcium while Ac2-26 (10 µm) induced cytosolic calcium levels (*p* < 0.01) Reduced ERK activation was observed in treatment with CsH (1 µm), CsA (5 µm) and in shAnxA1 cell line compared to the shControl cell line.	[30]
4-T1	Mouse mammary tumor	M2 marker mRNAs (Arginase 1 and PPARγ) in 4T1 conditioned media with the highest expression at 48 h, increases in a time-dependent manner after treatment (*p* < 0.05). Increase in CD206 expression was observed in 24 h post treatment with 4T1 conditioned media in AnxA1^+/+^ cells. (*p* < 0.05) At 24 h post treatment with AnxA1 peptide Ac2-26 (1 µm), high levels of Arg1 and low levels of IL12 mRNA were observed. (*p* < 0.05) 4-T1 CM had 3 fold higher levels of CCL5 compared with serum free media. AnxA1 mRNA and Arginase 1 (M2-marker) was upregulated in macrophages upon treatment with recombinant CCL-5 treatment (10 pg mL^−1^) (*p* < 0.05). Opposite result was observed, where the AnxA1 mRNA and Arginase 1 expression was prevented by using anti-CCL5. (*p* < 0.05) Arg1 mRNA expression levels from AnxA1^+/+^ BMDM were increased upon CCL5 treatment, whereas AnxA1^−/−^ treatment with CCL5 recombinant protein was unresponsive. (*p* < 0.05) The 4T1-CM or Ac2-26 peptides significantly induced phosphorylation by inhibiting the FPR2 antagonist (WRW4, 10 μg/mL^−1^) of ERK1/, AKT and NF-κB (p65).	[28]
MDA-MB 231	Human breast cancer	The mRNA and protein expression level of MMP-9 was decreased by 80% in a dose dependent manner after 24 h in cells transfected with 10nM and 30nM siAnxA1. AnxA1 siRNA reduced the proteolytic activity of MMP-9 in MDA-MB 231 cells The promoter activity of MMP-9 (−925/+13) in cells transfected with AnxA1 siRNA was downregulated by 2.2-fold in a dose dependent manner. Luciferase activity in cells transfected with the NF-κB reporter was decreased in a dose dependent manner by AnxA1 siRNA. AnxA1 siRNA reduced the DNA binding activities of NF-κB in a dose dependent manner.	[13]
MDA-MB 157, MDA-MB 436, HS578T, MDA-MB 468, BT549	Human breast cancer	All the TNBC cell line was found to have high level of EGFR, c-Met, and pAKT pathways compared to non-TNBC cell line. Phosphorylation of mTOR inhibition in AnxA1 knockdown clones was 35% and 37% in MDA-MB 436 and MDA-MB 468 respectively as compared to scramble shRNA control. (*p* < 0.01). Significant inhibition was observed in phosphorylation of ribosomal protein S6 (53% and 37% in MDA-MB 436 and 468 respectively) (*p* < 0.01) Not only that, inhibition in pEGFR levels was observed in MDA-MB 468 cell. (*p* < 0.01) AnxA1 knockdown cells showed increase in phosphorylation of AMPKα (almost 1.8-fold) (*p* < 0.01)	[20]
MDA-MB 231	Human breast cancer	High level of FPR1 mRNA was observed in MDA-MB 231 cells as compared to human monocytes. (4-fold) Whereby, FPR2 mRNA was higher in MDA-MB 231 cells as compared to MCF-7 cell line. (8-fold)	[27]
MDA-MB 231	Human breast cancer	Knockdown of AnxA1 in MDA-MB 231 cells reduced the NF-κB DNA binding.(*p* < 0.01) Such decrease in IκBα phosphorylation and degradation was also observed in AnxA1 knockdown cells. Treatment with TNF-α (10 ng/mL), stimulated IκBα phosphorylation, whereby cells transfected with anti-sense oligonucleotide (ASO) for 48 h showed no IκBα activation. MDA-MB 231 cells express high level of AnxA1, NEMO and RIP1. Silencing AnxA1 in MDA-MB 231 cells have disrupted the expression of NEMO and RIP1. MDA-MB 231 cells transfected with ASO and followed by treatment with TNF-α decreased the uPA mRNA expression. AnxA1 regulates cellular protein levels of CXCR4 (i) and also surface expression of CXCR4 (ii).	[25]
MDA-MB 231	Human breast cancer	AnxA1 knockdown cells exposed to TGF-β impaired the phosphorylation of Smad2 Knockdown of AnxA1 in MDA-MB 231 cells exposed with TGF-β for 30 minutes exhibited reduced translocation of Smad4 AnxA1 depleted cells exhibited a decrease in the activity of TGF-β/Smad3/Smad4-driven (CAGA)_12_. (*p* < 0.05)	[26]
BT-549, MDA-MB 231	Human breast cancer	DCST1-AS1 expression was not affected in AnxA1 knockdown cells (MDA-MB 231 and BT-549 cells) However, DCST1-AS1 knockdown cell lines (MDA-MB 231-sh and BT-549-sh) downregulated the expression of AnxA1 expression (*p* < 0.01) Significant decrease in AnxA1, Vimentin, SNAI1 and MMP-9, while increase in E-Cadherin were found in DCST1-AS1 knockdown cells (MDA-MB 231 and BT-549) TGF-β treated BT-549-sh cells (30 minutes) showed an impaired phosphorylation of Smad2 as compared to the negative control (BT-549 cells). However, when the DCST1-AS1 knockdown BT-549 cells were transfected with pcDNA3.1-AnxA1, and treated with TGF-β for 30 minutes, the results showed that AnxA1 has the potential to partially reverse the impaired phosphorylation of SMAD2 triggered by knockdown of DCST1-AS1	[33]

## Data Availability

Not applicable.
